# Personality Classification of Social Users Based on Feature Fusion

**DOI:** 10.3390/s21206758

**Published:** 2021-10-12

**Authors:** Xiujuan Wang, Yi Sui, Kangfeng Zheng, Yutong Shi, Siwei Cao

**Affiliations:** 1 Faculty of Information Technology, Beijing University of Technology, Beijing 100124, China; xjwang@bjut.edu.cn (X.W.); ytShi@emails.bjut.edu.cn (Y.S.); caosiwei@emails.bjut.edu.cn (S.C.); 2 School of Cyberspace Security, Beijing University of Posts and Telecommunications, Beijing 100876, China; kfzheng@bupt.edu.cn

**Keywords:** natural language processing, personality recognition, social text, multi-head self-attention, convolutional neural network, bi-directional long short-term memory network

## Abstract

Based on the openness and accessibility of user data, personality recognition is widely used in personalized recommendation, intelligent medicine, natural language processing, and so on. Existing approaches usually adopt a single deep learning mechanism to extract personality information from user data, which leads to semantic loss to some extent. In addition, researchers encode scattered user posts in a sequential or hierarchical manner, ignoring the connection between posts and the unequal value of different posts to classification tasks. We propose a hierarchical hybrid model based on a self-attention mechanism, namely HMAttn-ECBiL, to fully excavate deep semantic information horizontally and vertically. Multiple modules composed of convolutional neural network and bi-directional long short-term memory encode different types of personality representations in a hierarchical and partitioned manner, which pays attention to the contribution of different words in posts and different posts to personality information and captures the dependencies between scattered posts. Moreover, the addition of a word embedding module effectively makes up for the original semantics filtered by a deep neural network. We verified the hybrid model on the MyPersonality dataset. The experimental results showed that the classification performance of the hybrid model exceeds the different model architectures and baseline models, and the average accuracy reached 72.01%.

## 1. Introduction

Personality refers to the difference in thought pattern, emotion, motivation, and behavior characteristics of individuals [1], which has the basic characteristics of integrity, stability, uniqueness, and sociality. Personality test results are widely used in many fields such as personalized services, personalized medicine, sentiment analysis/opinion mining, and clinical psychology. Personality theory can be divided into six schools: psychoanalysis, traits, biology, humanism, behaviorism, and cognition schools. The most commonly used personality model is the Big Five [2], which is the most popular in trait schools. It describes personality from five aspects: openness (OPN), conscientiousness (CON), extraversion (EXT), agreeableness (AGR), and neuroticism (NEU).

Traditional methods of personality assessment often rely on interviews or self-report scales. This method requires a significant amount of manpower and material resources, but the feedback is limited in quantity and quality [3]. In recent years, deep learning has made significant progress in the field of natural language processing and has become more powerful in text modeling. Moreover, with the use of large-scale training data, the recognition errors caused by deep neural networks have been significantly reduced compared to traditional empiricist approaches.

The rapid development of the Internet and the popularity of social media tools, such as Facebook, microblogs, and Twitter, has made it easy for researchers to become interested in social network analysis. The development of automatic personality recognition has also been injected with great potential. In the computer age, it is easy to obtain rich data that are generated when people use terminal devices and carry out social network activities. Psychological research shows that there is a correlation between network data and personality characteristics [4], which reveals the user’s personal information, decision-making style, and ideological tendency. Therefore, the openness and accessibility of user text data make the corpus of personality classification tasks more abundant and provides convenience for personality modeling as well. Researchers usually collect posts from users at different stages and aggregate the scattered posts into a user personality profile for personality detection.

Current research methods use a single model to encode each post independently, which ignores the dependencies between posts, and the extracted features are not comprehensive enough to fully mine the personality information in user data. Another alternative approach is to combine scattered posts into sequences of arbitrary lengths for personality detection in a sequential or hierarchical coding manner [5,6]. However, human is a complex and variable complex, and the information contained in different text posts may contribute to different personality traits to different degrees.

Moreover, in the field of deep learning, to improve the accuracy of personality prediction, previous studies have linked the features extracted by deep neural network models with additional social network analysis (SNA) features or linguistic features. Moreover, personality detection models in existing works usually rely on increasing the depth of the network structure to extract semantic features in social texts.

In this paper, we propose a hierarchical hybrid model based on a self-attention mechanism, called HMAttn-ECBiL, consisting of HMA-CNN, HA-BiLSTM, and the original word embedding module, and the main contributions of this paper are as follows:

- HMA-CNN: we embed the multi-headed self-attention mechanism into the CNN architecture by dividing the text sequence into multiple regions to learn the local feature representation of each region in a cascade computation, and then gradually expand the region to model the global feature relationships in a hierarchical manner.
- HA-BiLSTM: we use the word attention mechanism to generate sentence-level feature representations. Then, we combine the scattered posts into multiple sequence fragments of the same length, and use Bi-LSTM and sentence-level attention mechanism to calculate the temporal characteristics of the captured text sequence and the contribution of different posts to personality traits.
- HMA-CNN, HA-BiLSTM, and word embedding multiple modules perform feature fusion in a parallel manner to compensate for the limitations of features extracted by a single model, maximize the use of rich semantic information of text data, and ensure the integrity and diversity of features, thus improving the efficiency and accuracy of personality classification tasks.

The rest of this paper is organized as follows. In Section 2, we discuss related work. Then, we elaborate on the mixed model for personality classification in Section 3. In Section 4, we present the experimental process and simulation results of the comparative experiment. Finally, in Section 5, conclusions are drawn and plans for future work are proposed by summarizing the model and experimental results.

## 2. Related Work

In recent years, studies in the field of psychology have found that individual differences can affect language usage habits, including the frequency of emotional vocabulary [1,4]. Thus, the text data generated by users in social media implies personality information. Two methods have been designed to establish an effective personality prediction system based on language features. One is the closed vocabulary based on predefined vocabulary categories, such as linguistic inquiry and word count (LIWC), structured programming for lingual cue extraction (SPLICE), and SNA. The other is the open-vocabulary approach implemented by a word-embedding model (e.g., Glove and Word2vec). The model can provide a unique word vector for each word in the corpus, and the word vector can represent semantic information and word spacing, so it is more flexible.

Most of the personality prediction methods use traditional machine learning algorithms to learn shallow features of text from user’s online activity data or personal profile information for classification tasks. Michael et al. [7] took advantage of the dataset of the myPersonality project to compare the performance of four machine-learning models and explored the correlation between linguistic features and personality characteristics. The results showed that the XGBoost classifier achieved the highest prediction accuracy of 74.2%. Moreover, a personality prediction system based on social network analysis features reached the best performance.

In the process of automated metaprogram detection and personality type prediction based on MBTI personality type indicators, Amirhosseini et al. [8] used a new machine learning method developed with the natural language processing toolkit and XGBoost. Han et al. [9] proposed a personality recognition model based on personality lexicon, which analyzed relationships between semantic categories of user microblogs and personality scores and used machine learning classifier for recognition task.

In recent years, end-to-end deep neural network architectures have become more powerful in text modeling, and have made significant progress in natural language areas such as text-based sentiment classification, speech recognition, machine translation, and opinion mining, yielding more accurate prediction results.

Convolutional neural networks (CNN) is one of the mainstream architectures that can extract n-gram of high-level features in local windows using different convolutional filters. Based on stream-of-consciousness essays, Majumder et al. [10] used a CNN model to extract feature vectors in a corpus in a hierarchical way, combined with document-level Mairesse features as the input of personality classifier. The experimental data showed that such a multilevel perceptron (MLP) had a higher classification accuracy than other classifiers.

However, CNN ignores word order and context information. Researchers try to model the time dependence between sentences by feeding the input back to the recursive neural network (RNN). Further, LSTM [11] was proposed to solve the problem of gradient disappearance and gradient explosion in RNN when the text sequence is too long. Sun et al. [12] introduced the latent sentence group concept to represent the abstract feature combination based on tightly connected sentence vectors, they combined Bi-LSTM with a CNN to recognize personality by utilizing text structure.

Based on the Big Five personality model, Tandera et al. [13] uses machine learning algorithms and deep neural networks to construct personality classification models. In addition, LIWC, SPLICE, and SNA features are used as different input features, and feature selection and resampling techniques are used as additional optional processes. Experiments show that the classification accuracy of deep neural network architecture is higher than that of machine learning algorithms. In view of the multimodality and heterogeneity of smartphone sensing data, Gao et al. proposed a deep neural network model to fuse multisource features [14], which performed the classification of Big Five personality in the manner of multitask learning. Experimental results showed that the performance metrics of the proposed approach significantly outperformed shallow machine-learning models.

One of the important technological breakthroughs in applying deep learning to natural language processing problems is the proposal of the attention model [15]. In the field of NLP, the attention mechanism may enable the model to select important information that needs attention based on the input and generated content [16] or generate soft alignment between the input and output to alleviate the problem of sequence change and difference in certain tasks [17] (for example, machine translation and text summarization) to enhance text modeling.

Xue et al. [5] designed a two-level hierarchical deep neural network model, AttRCNN, and proposed a variant of the inception structure based on a CNN. The lowest average prediction error was obtained by the approach using the concatenation of statistical linguistic features and the deep semantic features extracted by a hierarchical model. Lynn et al. presented a hierarchical sequence model that used message- and word-level attention to learn the relative weight of users’ social media posts to identify personality [6]. Experimental results demonstrated that models with message-level attention were superior to other baseline models, and the attention mechanism greatly improved the performance of personality prediction.

In order to better perform efficient parallel training and capture long-distance sequence features, Transformer [18] makes the architecture scale up and down with the training data and model size. The Transformer architecture is suitable for pre-training on a large text corpus and can perform well on specific tasks. Therefore, it has become the dominant architecture in the field of natural language processing and has achieved significant performance improvements in tasks such as natural language understanding [19], machine translation [20], and text generation [21,22].

Keh et al. [23] verified the use of a pre-trained language model to predict the classification accuracy of MBTI personality types and used fine-tuning techniques to adapt the BERT two-way converter model to corpus and language generation tasks. Jiang et al. [24] fused pre-trained context embedding (Bert and RoBERTa) and an attention neural network to construct a novel method of automatically identifying personality. The performance of this method on monologue essays is better than the latest results. In order to study the dependency between the personality information implied by scattered social media posts, and to solve the unnecessary post-order bias caused by any combination of posts, Yang et al. proposed a multi-document transformer named Transformer-MD [25], and on this basis, designed a dimensional attention mechanism to obtain the trait-specific representation of each personality dimension.

Furthermore, the feature fusion technology ensures the completeness and diversity of information, improves the performance of the model, and performs strongly in various tasks. Polap et al. [26] innovatively applies the bag-of-words mechanism to unconventional ship image classification tasks and uses convolutional neural networks to classify and capture keypoint features in local images, so that the results of ship classification are improved by 5% on the basis of classic methods. In addition, Nagaoka et al. [27] propose a convolutional neural network architecture that is sensitive to text scale. It extracts feature maps of different resolutions in multi-level convolutional layers and fuses text information features of different scales to prevent loss of information during the convolution process.

In order to explain the related work of the personality recognition task more clearly, in Table 1 we display the feature types and contributions of related models to compare their algorithm differences and performance. It can be seen that the previous work tends to improve the accuracy of personality classification by increasing the depth of the network or introducing external knowledge.

## 3. Materials and Methods

### 3.1. Personality Classification Model

**Figure 1:**
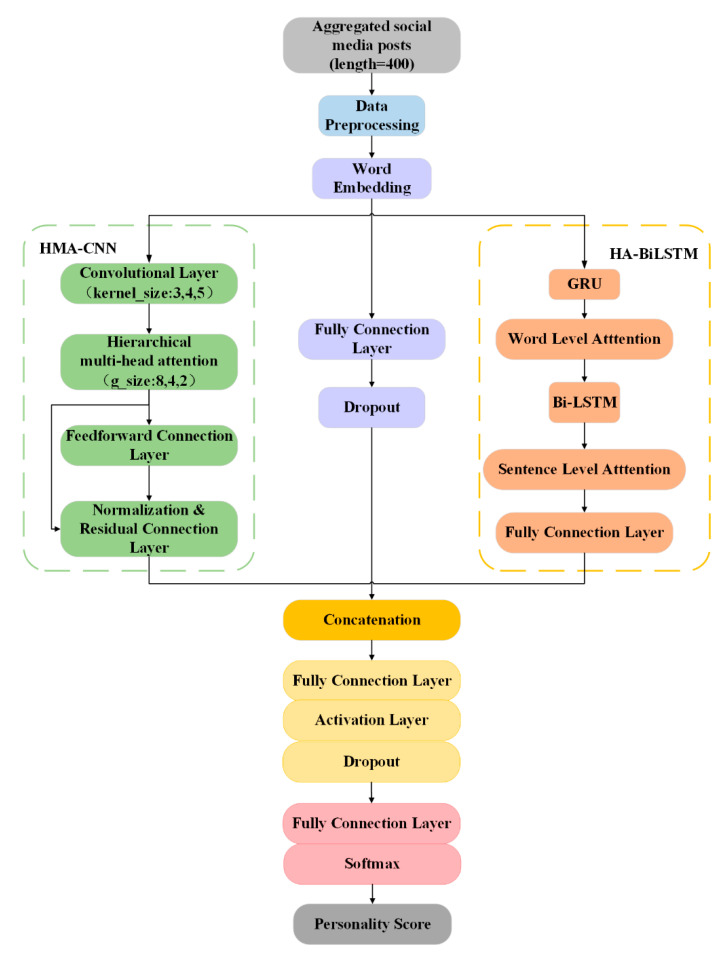
Model flowchart.

Figure 1 shows the hierarchical hybrid model based on the self-attention mechanism HMAttn-ECBiL, including three modules: the convolutional neural network HMA-CNN with embedded multi-head self-attention, the hierarchical attention mechanism combined with bidirectional long short-term memory network HA-BiLSTM and original word embedding module. The three modules perform feature fusion in a parallel manner, which makes up for the limitations of the features extracted by a single model, ensures the integrity and diversity of features, and finally realizes the personality classification of social network users based on the Big Five personality model. The model is elaborated as follows.

### 3.2. Data Preprocessing

To improve data quality and avoid dirty data, data preprocessing transforms the original dataset into an available and standard dataset before putting the data into model training. The preprocessing operation includes text segmentation, data cleaning, and data filling, such as the removal of stop words, English involves case conversion, removal of useless tags, and special symbols, etc.

The dataset is composed of text posts from 250 Facebook users. Social users tend to use informal languages and custom symbols to emphasize their emotions, such as “soooooo, HELP, ????, (* ∼ *)”, and so on. Although these special words are helpful to personality classification, they may bring great challenges to the training of the word embedding model. On the basis of maintaining semantic features as much as possible, our preprocessing process carries out the following operations: deleting repeated characters, case conversion, deleting redundant spaces to help model word segmentation, etc. Therefore, the above special words will be converted to “so, help, ?, (* ∼ *)”.

Because the amount of vocabulary in the NLP field is generally very large (i.e., reaching the level of millions), it is simple to express word vectors using one-hot representation. However, this generally causes dimensional disaster and memory waste. The word-embedding model can embed a high-dimensional space with the number of all words into a low-dimensional continuous vector space, and the data format makes it easy for the computer to process.

Word2vec [28] is a language model used to learn word-vector representations developed by Google in 2013. This model not only vectorizes all words but also measures word semantic similarity and lexical semantic analogy. The preprocessed dataset provides a unique and meaningful word sequence, and each word has a unique vector. We used a pre-trained Word2Vec model for word embedding, with a vector dimension of 300D for each word. The model initialized words to assign random weights and was able to learn word-embedding representations.

### 3.3. Feature Extraction

#### 3.3.1. HMA-CNN

**Figure 2:**
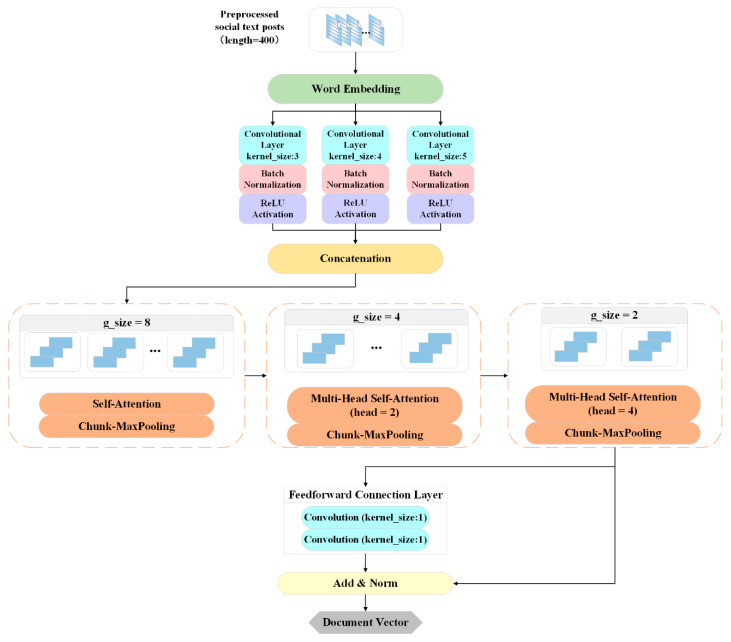
Architecture of HMA-CNN module.

The detailed architecture of the HMA-CNN module is shown in Figure 2. First, we take the word vector obtained by data preprocessing of a fixed-length text post and use n convolution kernels of different sizes in the convolution layer to extract the local features of the text data. Subsequently, we aggregate the n-gram features and divide them into different area sizes and input them into the multi-head self-attention mechanism (MHSA), and learn the local feature representation of each area in a cascade calculation method.

We reduce the number of partitions step by step, and gather local features at the same time, so as to model the global feature relationship in a hierarchical manner. In addition, the feedforward connection layer is used to deepen the degree of fitting of the attention mechanism to semantic features. Finally, in order to normalize the value within a reasonable range and prevent the model performance from degrading as the number of network layers deepens, we added a normalization operation and a residual connection block at the end of the HMA-CNN module. The following is a detailed description of the convolutional layer and (H-MHSA).

- Convolutional layers

One-dimensional convolution uses fixed-size convolution kernels to slide over the sequence and detect features in different positions. The maximum length of the aggregated user posts is denoted *max_length = L*, and *k* is defined as the length of the convolution kernel. Then, for each position *j* in the sentence, there is a window vector $wd_{j}$ and *k* consecutive word vectors, $j=1,2,\ldots \frac{k}{L}$. Each word vector is 300D; that is, *d* = 300. Let $x_{j}\in R^{d}$ be the d-dimensional word vectors for the *j*th word in the sentence, the sentence is marked with $x\in R^{L\times d}$, and the window vector is represented as follows:

$$wd_{j}=\left[x_{j},x_{j+1},\cdots ,x_{j+k-1}\right]$$

where $wd_{j}$ is a vector matrix composed of *k* word vectors. A feature map $h\_map_{j}$ is obtained by convolution operation of window vector $wd_{j}$; the calculation process is shown in Equation (1):

(1) $$h\_map_{j}=f\left(wd_{j}\cdot conv+b\right)$$

where $conv\in R^{k\times d}$ is defined as the convolution operation of the filter in a valid way, · is element-wise multiplication, *b* is a bias term, and *f* is a nonlinear function that can be sigmoid, a hyperbolic tangent, and so on. In this work, we selected ReLU as the nonlinear function. In general, the initial value of the bias unit took a random value that was automatically updated by back-propagation when training the model and was adjusted to the convergence of the loss function. Hence, $h\_map\in R^{L-k+1}$ represented the feature mapping of all window vectors in the entire sentence obtained by the convolution operation.

In this study, we used *n* convolution kernels of different sizes to obtain n-gram features, *n* = 3, $k=\left(k_{1},k_{2},k_{3}\right)=\left(3,4,5\right)$, and the numbers of convolution kernels with different sizes were *num_filters*. To ensure that the output vector of the convolution operation of each size is consistent with the input dimension, “SAME” is selected as the padding way. After the convolution operations, we appended the feature h_map obtained by *num_filters* convolution kernels of the same window size $k_{i}$ together to obtain the feature $P_{k_{i}}$:

$$P_{k_{i}}=\left[h\_map_{1};h\_map_{2};\ldots ;h\_map_{num_{filters}}\right]$$

where the semicolon represents the concatenation of column vectors. In addition, the convolution kernels of different window sizes are spliced together again, and the features obtained after splicing are represented as *Conv_output*:

$$\mathrm{Conv}\_\mathrm{output}=\left[P_{k_{1}};P_{k_{2}};P_{k_{3}}\right]$$

- H-MHSA

CNN only pays attention to the mutual influence of word pairs in the local window and cannot take into account all word pairs. Therefore, we add a multi-head self-attention structure (MHSA) to HMA-CNN to extract global features of different representation subspaces. However, if the input sequence is too long, that is, the vector dimension is too large, compared with a shorter text sequence, the relevance of the same word pair extracted by MHSA will be diluted by other words and decrease naturally. In addition, MHSA is inefficient due to high computational complexity.

In the H-MHSA structure, we extract the n-gram feature vectors *Conv_output* from a fixed-length text post through the convolutional layer and split them into multiple regions in the dimension of the sequence length, and then use MHSA to calculate the words in each partition dependency, where *g_size* marked in Figure 2 is the number of regions divided by each layer. Then, the small areas are gradually merged into larger areas, and local feature representations are also gathered. Subsequently, the self-attention is calculated again in the new partition, and the global characteristics of the sequence are naturally modeled in a hierarchical manner. Therefore, H-MHSA can more accurately capture the interaction of word pairs and the dependency between posts and reduce the dimensionality of the input vector with the help of partitioning and layering, thereby improving and reducing the computational complexity of MHSA.

Suppose that for a certain layer of the MHSA structure, the height of the input feature vector $X\in R^{L_{0}\times D_{0}}$ is the number of tokens $L_{0}=400$, where each token is characterized using a vector of dimension $R^{1\times D_{0}}$. Then, we divide the whole input vector into multiple regions according to the set number of partitions *g_size*, and the height of the sequence features in each region is $G_{0}=\frac{L_{0}}{g\_size}$. Thus, the input feature vector *X* is reconstructed as $X^{\prime }\in R^{\left(\frac{L_{0}}{G_{0}}\times G_{0}\right)\times D_{0}}$, and we obtain the query, key, value:

(2) $$Q,K,V=X^{\prime }W^{q},X^{\prime }W^{k},X^{\prime }W^{v},$$

Among them, $W^{q},W^{k},W^{v}\in R^{D_{0}\times d\_model}$, respectively, represent the learnable parameters of the query, key, and value in Transformer [18]. We use MHSA to calculate the self-attention within the partition to obtain a new text representation $A\in R^{\left(\frac{L_{0}}{G_{0}}\times G_{0}\right)\times d\_model=L_{0}\times d\_model}$ as follows:

(3) $$A=softmax(QK^{T}/\sqrt{d})V$$

where $\sqrt{d}$ represents approximate normalization. For the sake of simplicity, we have omitted the expression of the calculation method of multiple heads.

In order to simplify feature characterization, avoid information redundancy, and further reduce the height of the area block to improve computational efficiency, we added a max pooling layer after the MHSA structure of each layer, and the pooling operation uses the Chunk-MaxPooling method. The basic idea of Chunk-MaxPooling is to cut the feature vector into several segments, and then obtain a maximum feature value in each segment. We divide the text representation A obtained by the MHSA structure into L0/2 fragments composed of 2 tokens, and then obtain the down-sampled new text representation $A^{\prime }\in R^{\frac{L_{0}}{2}\times d\_model}$.

The feedforward connection layer is composed of two convolutional layers, so the convolution operation is roughly the same, so we will not repeat it here. After adding normalization and residual connection to the output vector of the feedforward connection layer, the final document vector *CD* of the HMA-CNN module is obtained:

(4) $$CD=norm\left(FeedForward\left(A^{\prime }\right)+A^{\prime }\right)$$

#### 3.3.2. HA-BiLSTM

In the HA-BiLSTM module, we use a hierarchical attention mechanism to encode social user posts into feature representations that can be used to predict individual personality. CNN can extract local spatial or short-term structural relationships, but it has poor ability to extract features for sequence data. Although the MHSA in Transformer can extract the long-distance dependence of the entire text sequence, it is also insensitive to the text order due to the lack of location information. In response to this problem, we added bi-directional long short-term memory (Bi-LSTM) to the model to obtain contextual information and better capture the bidirectional semantic dependence of social text sequences. Among them, compared with recurrent neural network (RNN), LSTM adds a gate mechanism to filter information, and to a certain extent avoids the problems of gradient disappearance and gradient explosion.

**Figure 3:**
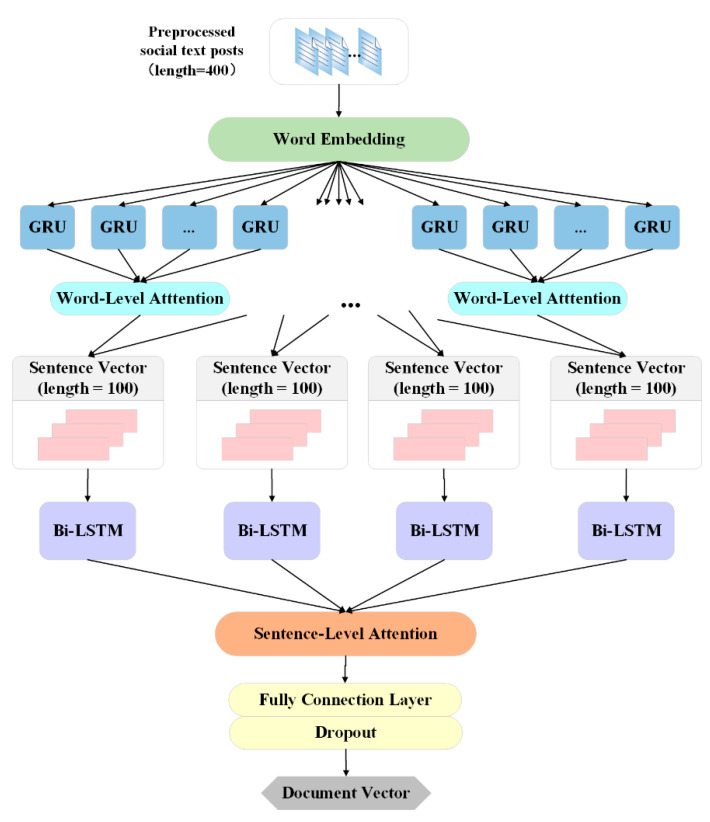
Architecture of HA-BiLSTM module.

First, we use gated recurrent unit (GRU) encoding for the word embedding vector of each word in the post and use the word attention mechanism to form a sentence set feature representation. Then, we combine the scattered posts into multiple sequence fragments of the same length, use Bi-LSTM to extract the temporal features of the text, and then use the sentence-level attention mechanism to calculate the personality information carried in different posts, and capture the sequence fragments dependency. In order to map the sentence-level feature representation of the hierarchical attention output into a document vector and avoid overfitting, we added a fully connected layer and a dropout layer at the end of the module. The detailed architecture of the HA-BiLSTM module is shown in Figure 3.

- Word-Level Attention

The attitude expressed in a sentence is not determined by all the words together, such as “I met a cute cat on the way to school”. In this sentence, only the word “cute” expresses emotion and attitude, while on the “Way to school” is only a statement of facts, so if we analyze a person’s emotions and personality based on the text, we must pay more attention to emotional words such as “cute”.

Similarly, among the many posts published by social users, not all texts have a decisive effect on the user’s personality. We should encode the most valuable information as a representation of personality characteristics. Therefore, we use the word-level attention machine to learn the words in the text sequence that are highly associated with personality to encode them as sentence-level representations and use sentence-level attention to emphasize the information related to the personality to aggregate into the overall document vector.

For example, a user published *n* posts, the *i*-th post consists of *M* words, and each word $e_{j}^{i}$ generates a hidden state $h_{j}^{i}$ through GRU:

(5) $$h_{j}^{i}=GRU(e_{j}^{i})$$

Then, we apply the word attention mechanism to the generated sequence of hidden states:

(6) $$d_{j}^{i}=tanh(W_{e}h_{j}^{i}+b_{e}),$$

(7) $$\alpha_{j}^{i}=\frac{\exp(d_{j}^{i\;T}d_{context})}{\sum_{m=1}^{M}\exp(d_{m}^{i\;T}d_{context})},$$

(8) $$v_{i}=\overset{M}{\underset{m=1}{\sum }}\alpha_{m}^{i}h_{m}^{i}$$

where $d_{context}$ is a learned context vector for word-level attention, exp is the exponential function,$\;\alpha_{j}^{i}$ is the attention weight obtained by the hidden vector corresponding to the j-th word in the i-th post, $W_{e}$ is the weight matrix, and $b_{e}$ is the bias coefficient. The initial values of the two are generally random values. This value is automatically updated through backpropagation when training the model. Therefore, according to the weight corresponding to each word, the feature representation $v_{i}$ of the *i*-th post is obtained.

- Bi-LSTM Layer

**Figure 4:**
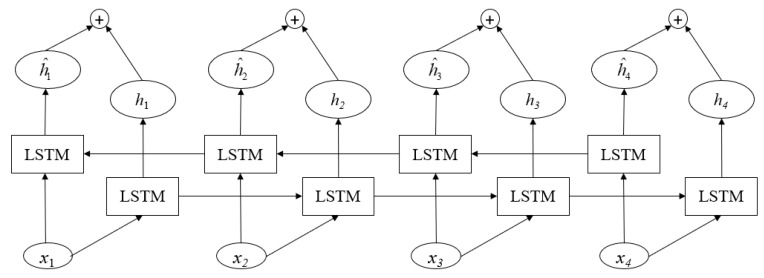
Structure of Bi-LSTM.

Bi-LSTM is a combination of forward LSTM and backward LSTM, which solves the problem that uni-directional LSTM cannot encode information from back to front. Bi-LSTM adds a delay between the input and target and several time steps to the network for joining the future context information. Thus, it can really use the context information to predict the output. Therefore, we integrated Bi-LSTM instead of LSTM into the model to better capture the bi-directional semantic dependency of social text sequences. The network structure of Bi-LSTM is shown in Figure 4.

In order to capture the long-distance temporal characteristics of user posts, we divide *R* post representations encoded by word-level self-attention mechanism into a group $\left[v_{1},v_{2},\cdots ,v_{R}\right]$, and aggregate them into *C* new sequence fragments $V_{c}$ of length 100, where c=1,2,⋯,C. Since the sequence length processed by Bi-LSTM is limited, the length of sequence fragments should not be too long, so as to avoid the disappearance of the gradient. Each sequence fragment $V_{c}$ selectively forgets or remembers the information in the context cell state through Bi-LSTM, so that information useful for cell state calculations can be transmitted, while useless information is discarded, and the hidden layer state $h_{t}{}^{c}$ will be output at each time step. The word vector of the input layer will be calculated in both forward and backward directions, and the hidden state of the final output will be connected to obtain a new sentence vector, as shown in Equation (9).

(9) $$s_{c}=concat\left(h_{forward}^{c},h_{backward}^{c}\right)$$

- Sentence-Level Attention

After obtaining the sentence vector, we can use the sentence-level attention mechanism to encode the sequence segment into a document vector *u*. The encoding process is similar to that of the word attention mechanism, as shown in Equations (10)–(12):

(10) $$r_{c}=\tanh(W_{s}s_{c}+b_{s}),$$

(11) $$\beta_{c}=\frac{\exp(r_{c}{}^{T}r_{context})}{\sum_{o=1}^{C}\exp(r_{o}{}^{T}r_{context})},$$

(12) $$u=\overset{C}{\underset{o=1}{\sum }}\beta_{o}s_{o}$$

where $r_{context}$ is a learned context vector for sentence-level attention, $\beta_{o}$ is the attention weight obtained by the sentence vector of the *c*-th sequence segment. The document vector *u* is obtained by a weighted combination of all sentence vectors, and the final user personality characterization *LD* of the HA-BiLSTM module is obtained through the fully connected layer and the dropout layer.

### 3.4. Feature Fusion and Classification

The vector matrix of social text data processed by the Word2Vec model can extract different types of deep semantic information through architectures such as CNN, Bi-LSTM, and H-MHSA, but it inevitably loses the characteristics of the original text features.

Therefore, to make up for the lost semantics of the original matrix, we use the concat() function to concatenate the document vector *CD* encoded by the HMA-CNN module, the document vector *LD* encoded by the HA-Bi-LSTM module, and the original word vector data_dense after the nonlinear transformation of the FC layer according to the column vector to get fusion features oc, as shown in Equation (13):

(13) $$oc=concat\left(CD,LD,data_{dense}\right)$$

Furthermore, in order to extract the associations between multiple features and map the fusion features to the output space, we added a fully connected layer composed of dense_unit hidden layer neurons and an activation layer to fit oc. In addition, we use dropout operations to avoid feature redundancy and overfitting. Finally, we encode the user’s scattered posts as a personality representation pred for the prediction task.

In addition, to avoid overfitting and reduce feature redundancy in the training process of the deep-learning network, we used dropout to process the fusion feature. For neural network units, discarding them temporarily from the network according to a certain probability can weaken the joint adaptability of neuron nodes. After cross-verification, the effect was best when the hidden-node dropout rate (range of 0–1) was set to 0.5.

In this study, personality recognition was based on the Big Five personality model. The five types of personalities were not mutually exclusive, and each personality was a binary value: yes/no (0/1), so it belonged to the multilabel classification problem. When designing the classification model, we transformed the multilabel classification into five binary classification problems and then used multiple single-label classifiers to carry out the processing. The single-label classifier selected the normalized exponential function softmax(), which could “compress” an M-dimensional vector z with any real number into another M-dimensional real vector θ(z), so that the range of each element was between 0 and 1; the sum of all elements was 1. In our work, M should be the category quantity *class_num*=2. It is defined in Equation (14):

(14) $$\theta {\left(z\right)}_{a}=\frac{e^{z_{a}}}{\sum_{m=1}^{M}e^{z_{m}}},a=1,\ldots ,M,z=\left(z_{1},\ldots ,z_{M}\right)\in {\mathbb{R}}^{M}$$

where $\theta {\left(z\right)}_{a}$ is the probability that sample z belongs to the *a*th class. The samples $z\in R^{M}$ are defined in Equation (14) (i.e., *M* = 2). Because the function softmax() is used as a binary classifier, the output value $\theta \left(z\right)=(\theta {\left(z\right)}_{1},\theta \left(z{)}_{2}\right)$ should be similar to the format of [0.88, 0.12], and the maximum probability value is determined as the final predicted value. Therefore, the input value should also be a two-dimensional value for the purpose of meeting the input requirements of softmax function binary classification. We performed dot product operation on the fused feature $pred\in R^{dense3\_unit}$ and the weight matrix $wc\in R^{dense3\_unit\times 2}$, and added the corresponding bias coefficient $bc\in R^{2}$.

(15) $$z^{i}=pred\cdot wc^{i}+bc^{i},i=1,2,3,4,5$$

Superscript *i* represents the feature vector and super-parameter of the *i*th personality tag in Big Five personality. With the change of wc and bc in the progress of back-propagation, the output probability of softmax() was adjusted to improve the classification accuracy.

In order to explain the model proposed in this article more clearly, the two important modules in our Algorithms 1 and 2 are displayed in pseudo-code form.

**Algorithm 1** HMA-CNN**Input:** social post text ∈Training Set initialized with Word2Vec**Output:** document vector *CD*1:  **for**
*k* = 1,2, …, kernel_num **do**2:   $X_{k}$←$\;\mathrm{Conv}2d(X_{k-1}$$,\;kernel_{k})\;//\mathrm{calculate}\;\mathrm{convolution}\;\mathrm{layer}\;\mathrm{with}\;k-\mathrm{th}\;\mathrm{kernel}$3:   $X_{k}$←$\;\mathrm{BN}(X_{k})\;//\mathrm{batch}\;\mathrm{normalization}$4:   $X_{k}$←$\;\mathrm{ReLu}(X_{k})\;//\mathrm{nonlinear}\;\mathrm{activation}$5:  **end for**6:  $A_{0}$$=\;\left[X_{1};\ldots ;X_{k}\right]\;//\mathrm{concatenate}\;k\;\mathrm{results}\;\mathrm{for}\;\mathrm{different}\;\mathrm{convolution}\;\mathrm{kernel}$7:  **for** g in g_size **do**           //g_size = [8, 4, 2]8:   A ←MHSA(A,g)      //process with Multi-Head Self-Attention9:   CD=norm(FeedForward(A)+A)//calculate the result of the last loop10:  **end for**

**Algorithm 2** HA-BiLSTM**Input:** social post text ∈Training Set initialized with Word2Vec**Output:** document vector *LD*     //output the personality representation1:  **for**  *i* = 1,2, …, post_num **do**2:   $\mathrm{for}\;j=1,2,\text{…},\;{\mathrm{post}}_{i}\_\mathrm{length}\;\mathrm{do}$3:     $h_{j}^{i}\leftarrow GRU(e_{j}^{i})\;//\mathrm{get}\;\mathrm{hidden}\;\mathrm{state}\;\mathrm{to}\;\mathrm{each}\;\mathrm{word}\;$4:   $v_{i}\leftarrow$$\;\mathrm{Word}-\mathrm{Level}\;\mathrm{Att}(h_{j}^{i})\;//\mathrm{get}\;\mathrm{post}\;\mathrm{feature}\;\mathrm{with}\;\mathrm{Word}-\mathrm{Level}\;\mathrm{Attention}$5:   **end for**6:  **end for**7:  $V=\mathrm{regroup}([v_{1},v_{2},\ldots ,\;v_{post\_num}])\;//\mathrm{divide}\;\mathrm{into}\;\mathrm{groups}\;\mathrm{of}\;\mathrm{size}\;C$8:  **for** *c =* 1,2,…, *C*
**do**9:   $s_{c}$$=\mathrm{Bi}-\mathrm{LSTM}(V_{c})\;//\mathrm{process}\;\mathrm{with}\;\mathrm{Bi}-\mathrm{LSTM}$10:  **end for**11:  $S=[s_{1},s_{2},\ldots ,\;s_{c}]$12:  *LD* = Sentence-Level Att(*S*)   //calculate with Sentence-Level Attention

## 4. Experiment and Analysis

### 4.1. Dataset

The experimental data used in this study are from the myPersonality dataset [29], which includes the social data from 250 Facebook users with approximately 10,000 statuses, in which the given personality label is based on the Big Five personality model. It is a complete dataset of social network users, including user text information and external information (such as the time of posting, network size, and so on). The research used plain-text data of myPersonality named myPersonality_text, removing the user’s external information. We divided the processed dataset into training and test sets into a 9:1 ratio.

### 4.2. Evaluation Metrics and Parameter Settings

We selected accuracy and F1 score as evaluation indicators of the experimental results, and the classification accuracy Acc was calculated as shown in Equation (16):

(16) $$Acc=\frac{TP+TN}{TP+TN+FP+FN}$$

We also used the F1 score to measure the accuracy of the binary classification model. It considered both the precision and recall of the classification model, which could be regarded as a harmonic average of the model accuracy and recall rate, with a maximum value of 1 and a minimum value of 0. The formula is defined in Equations (17)–(19):

(17) $$precision=\frac{TP}{TP+FP’}$$

(18) $$recall=\frac{TP}{TP+FN’}$$

(19) $$F1\_score=2\times \frac{precision\times recall}{precision+recall}$$

In actual training, we divided the dataset into several batches of size *batch_size* and calculated the accuracy and loss function of *batch_size* data. In Equation (16), *TP* is the number of actual positive cases in a batch of data that is divided into positive cases by the classifier, *TN* is the number of actual negative cases that is divided into negative cases by the classifier, *FP* is the number of actual negative cases that is divided into positive cases by the classifier, and *FN* is the number of actual positive cases that is divided into negative cases by the classifier; the positive-case label value is 1 and the negative-case label value is 0.

In our experiment, we trained the network with 50 epochs using cross-entropy loss function and Adam optimizer. We observed from the experimental results that with the increase of the number of iterations, the performance of the model in the training set is increasingly better, but it has not improved in the test set, that is, the model has an overfitting phenomenon. Therefore, we control the number of epochs to 50 and add a dropout operation to improve the generalization ability of the model. Due to the limitation of dataset size, setting batch_size to 32 and learning rate to 0.001 is the best combination. In addition, when the number of hidden layers in the fully connected layer is set to 128, the model achieves the best performance. If the number of parameters is too large, the model cannot adjust the parameters to the optimal value in back-propagation. The optimal values of more parameters are shown in Table 2.

### 4.3. Comparative Experiment on Length of Text Sequence

A correlation exists between user’s different posts on the social network, and different posts may express their views on the same thing. In addition, the aggregation of different posts into a whole also ensures that there is enough sequence information to help the model obtain stable personality characteristics. The sequence is too short to give full play to the advantage of MASA and Bi-LSTM in capturing long-distance dependencies, and the number of aggregated posts is too low to capture the dependencies between scattered posts. If, however, the text sequence is too long, the processing capacity of the model is limited, and the model focuses on memorizing a large amount of input information. On one hand, it will lead to a decline in the modeling ability to combine the predictive knowledge of different input vectors. On the other hand, it may also lead to the vanishing gradient problem in the process of back-propagation, weakening the reliability of the model and leading to performance degradation. Therefore, we set different text-sequence lengths to explore the influence of sequence length on the effect of the model.

We gathered the user posts with the same ID together, setting the text length to 200, 400, and 600 separately, and the fusion features were composed of original word features and document vectors extracted by the HMA-CNN and HA-BiLSTM. The experimental results are shown in Table 3. When the text length was 200, the average accuracy was the lowest at 63.16%. We increased the sequence length by 200, and the model accuracy and F1 score were improved. However, when the sequence length reached 600, the overall performance of the model followed a downward trend, and the average accuracy of all personalities decreased by approximately 6% compared with the sequence length of 400. According to the analysis, the classification effect was the best when the text length was 400, average classification accuracy was 72.01%, highest accuracy of open personality was 84.57%, and F1 score was 0.91.

### 4.4. Comparative Experiment of Different Model Architectures and Baseline Models

In order to verify the impact of different modules on the accuracy of personality classification, we constructed five models composed of different modules, as shown in Table 4.

**Figure 5:**
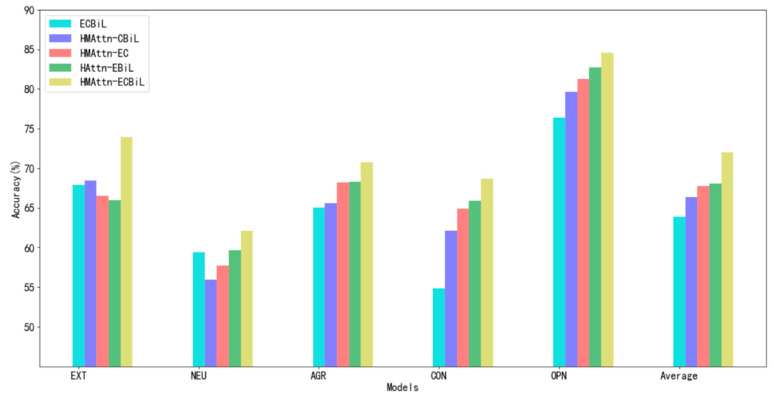
Classification accuracy with different model architectures.

Figure 5 gives a clear comparison of different models. Compared with the ECBiL model composed of the original CNN and Bi-LSTM, both the HMA-CNN module and the HA-BiLSTM module have a positive impact on the results of the personality classification task, because they captured the dependencies among scattered posts in different ways.

Moreover, the average classification accuracy of the HAttn-EBiL model is about 2% higher than that of the HMAttn-EC model. Therefore, we believe that it is necessary to calculate the contribution of different posts and different words in the posts to the user’s personality. HA-BiLSTM assigns different weights to words in different positions and different posts in a hierarchical manner and quickly filters out information that is more critical to the current task objective from a large amount of information, while the HMA-CNN module only extracts the aggregated information. Contextual dependence between information at different locations in the posts.

In addition, we also noticed that in addition to the ECBiL model, the HMAttn-CBiL model without the original embedding module achieved the lowest peak. It can be seen that with the increase in the number of network layers, the semantic features learned by the model become more diversified and abstract, while also inevitably filtering out some semantic features. The addition of the embedding model makes up for the original semantics of the global sequence features extracted by the HMA-CNN and HA-BiLSTM modules, thereby improving the classification accuracy. It is worth mentioning that the feature extraction process of the original embedding module must take operations such as dropout, regularization, and early stopping to avoid over-fitting.

In this research, HMAttn-ECBiL combined the features extracted from the word embedding module, HMA-CNN, and HA-BiLSTM modules to encode the personality representation of social users, and then the softmax function is used for the classification task. We compared personality recognition tasks with extracting text features using a single model, which is also based on the MyPersonality dataset. The experimental data comparison is shown in Table 5. The results showed that our hybrid model, HMAttn-ECBiL, achieved the highest personality classification accuracy, with an average classification accuracy of 72.05%. In the hybrid model, the accuracy of the five types of personality was more than 62%. Specifically, the classification performance for OPN was the best, with an accuracy of 84.57% and an F1 score of 0.92.

The classification accuracy of every trait in Big Five personality was different in all models. In addition to average accuracy, performance accuracy for OPN and AGR performed with the hybrid model HMAttn-ECBiL also scored higher than the baseline model using additional linguistic features or social network analysis features. Compared with the baseline model, the personality recognition accuracy of the proposed hybrid model was improved by 3–20%. In addition, the hierarchical model of word- and message-level attention [6] proposed by Lynn et al. was selected as the control group, HMAttn-ECBiL outperformed the hierarchical model in the accuracy of both five personality traits and average value.

The comparison of experimental data proves the superiority of our hybrid model. HMA-CNN and HA-BiLSTM encode different types of user personality representations in a partitioned and hierarchical manner. It carries the key semantic information related to personality information and the dependency between scattered posts. Therefore, the integration of multiple deep-learning technologies and an original word-embedding vector maximized the mining of text information both horizontally and vertically, thus increasing the depth and width of the network model and ensuring the integrity of semantic features. As a result, the classification performance was greatly improved.

## 5. Conclusions

Personality recognition is widely used in personalized recommendation, intelligent medicine, natural language processing, and other fields. At the same time, the great advantage of deep neural networks in text modeling promotes the development of classification tasks. In this paper, we proposed a hierarchical hybrid model based on a self-attention mechanism, called HMAttn-ECBiL, which was composed of HMA-CNN, HA-BiLSTM, and original word embedding module. On the one hand, HMA-CNN learned the global features in text data in a hierarchical cascade way. The division of sequence regions made the extracted semantic information more accurate and reduced the computational complexity of MHSA. On the other hand, HA-BiLSTM used different levels of attention mechanism and Bi-LSTM to capture the long-distance dependence and sequential characteristic in aggregated posts. It is worth mentioning that compared with HMA-CNN, HA-BiLSTM can focus on the key information for personality traits, thus greatly improving the classification accuracy.

Moreover, the addition of the word embedding model made up for some original semantics filtering by HMA-CNN and HA-BiLSTM modules, so as to ensure the integrity and diversity of features. The integration of multiple deep-learning technologies increased the depth and width of the network, making more effective use of text information. Compared with the baseline model constructed by different model architectures and single deep learning techniques, the hierarchical hybrid model based on self-attention mechanism HMAttn-ECBiL achieved the new state-of-the-art results in personality classification.

The informatization society and Big Data era have resulted in the hiding of personality privacy in all kinds of network-space text data. Based on the openness and accessibility of text data, the adoption of machine-learning algorithms and deep-learning models can effectively obtain a user’s personality information which becomes one of the most important channels for the leakage of personality privacy as well. Thus, in planned future studies, the protection of personality privacy will be a crucial research direction. Starting with the source of the weakness, we will analyze the principle of the leakage in personality privacy and then transform the text data, thereby reducing the personality privacy in the text data and blocking an attacker from analyzing the personality privacy in the data.

## Figures and Tables

**Table 1:** Brief description and comparison of important personality-detection models. (Sorted by year of publication).

Model	Dataset	Approach	Feature Type	Remarks
Majumder et al. CNN [10]	Stream-of-consciousness essays	Deep-learning technique, hierarchical modeling	Semantic features extracted by CNN, document-level stylistic features	Average accuracy: 62.68%
Tandera et al. LSTM + CNN 1D [13]	myPersonality	Deep learning + resampling technique, hierarchical modeling	Features extracted by combining LSTM and 1D CNN	1. Different language features and resampling techniques were used to set up different scenes. 2. Average accuracy: 62.71%.
Michael et al. SNA + XGBoost [7]	myPersonality	Machine-learning technique	SNA features	1. Study illustrated that a correlation exists between user personality and social network interaction behavior. 2. XGBoost classifier with SNA features can achieve highest prediction accuracy of 71.00% compared with linguistic features.
Xue et al. AttRCNN [5]	myPersonality	Deep-learning technique, hierarchical modeling	Deep semantic features extracted from AttRCNN, statistical linguistic features vectors	Average mean absolute error (MAE): 0.42768.
Lynn et al. Sequence Networks + Attn [6]	Facebook status posts of 68,687 users	Deep learning technique, hierarchical modeling	Word- and message-level attention feature representation	Model based on message-level attention achieved the best average accuracy: 54.98%.
Han et al. Random Forest [9]	Microblogs	Machine-learning technique	Personality lexicon combined keywords of microblogs and external knowledge	1. Personality explanation model proposed to analyze relationships between text features of user microblogs and personality scores. 2. F1 score: 0.737
Keh et al. [23] Bert	MBTI personality datasets	Deep learning technique	semantic features extracted from Bert	1. Accuracy: 0.47 2. A fine-tuned BERT model was used for personality-specific language generation.
Yang et al. Transformer-MD [25]	MBTI personality datasets	Transformer, MLP	Aggregated post feature representation dimension-specific representation	1. Transformer-MD captures the dependencies between social text posts without introducing post-order bias. 2. The dimensional attention mechanism is designed to capture the impact of different dimensions of posts on each personality trait.
HMAttn-ECBiL	myPersonality	Deep-learning technique, hierarchical and parallel modeling	Fusion features: word vector and two kinds of document vectors	1. Hybrid model combines the original word-embedding vector and the proposed modules including HMA-CNN, HA-BiLSTM. 2. Highest average classification accuracy: 72.01%.

**Table 2:** Overall parameter settings.

Parameter	Value
batch_size	32
learning_rate	0.001
dropout rate	0.5
embedding_size	300
max_length	400
num_filters	128
g_size in HMSA	[8,4,2]
number of head in H-MHSA	[1,2,4]
hidden_size	128
dense_unit	256
hidden activation	ReLU

**Table 3:** Classification accuracy and F1-score comparison of fusion features extracted from different length sequences.

Model	Sequence Length	EXT	NEU	AGR	CON	OPN	Average Accuracy
HMAttn-ECBiL	200	62.09%/0.73	53.04%/0.69	66.03%/0.79	61.23%/0.72	73.43%/0.86	63.16%
	400	73.94%/0.79	62.14%/0.76	70.74%/0.83	68.65%/0.81	84.57%/0.91	72.01%
	600	65.05%/0.69	55.02%/0.66	66.11%/0.76	62.82%/0.75	79.68%/0.89	65.74%

**Table 4:** Models composed of different modules.

Model.	Module
ECBiL	CNN, Bi-LSTM + original word embedding module
HMAttn-EC	HMA-CNN + original word embedding module
HAttn-EBiL	HA-BiLSTM + original word embedding module
HMAttn-CBiL	HMA-CNN + HA-BiLSTM
HMAttn-ECBiL	HMA-CNN, HA-BiLSTM + original word embedding module

**Table 5:** Comparison of classification accuracy between hybrid and single models on MyPersonality dataset.

Model	EXT	NEU	AGR	CON	OPN	Average Accuracy
CNN [10]	58.09%	59.38%	56.71%	57.30%	62.68%	58.83%
LSTM + 1D CNN [13]	71.05%	58.97%	50.00%	57.69%	75.86%	62.71%
SNA + XGBoost [7]	78.60%	68.00%	65.30%	69.80%	73.30%	71.00%
Sequence Networks + Attn [6]	55.20%	54.10%	50.90%	52.10%	62.60%	54.98%
HMAttn-ECBiL	73.94%	62.14%	70.74%	68.65%	84.57%	72.01%

## Data Availability

Restrictions apply to the availability of these data. Data was obtained from David Stillwell and are available at http://mypersonality.org (accessed on 27 August 2021) with the permission of David Stillwell.
